# Investigation of target sequencing of SARS-CoV-2 and immunogenic GWAS profiling in host cells of COVID-19 in Vietnam

**DOI:** 10.1186/s12879-022-07415-1

**Published:** 2022-06-19

**Authors:** Tham H. Hoang, Giang M. Vu, Mai H. Tran, Trang T. H. Tran, Quang D. Le, Khanh V. Tran, Tue T. Nguyen, Lan T. N. Nguyen, Thinh H. Tran, Van T. Ta, Nam S. Vo

**Affiliations:** 1 Center for Biomedical Informatics, Vingroup Big Data Institute, 458 Minh Khai Street, Hai Ba Trung, Hanoi, Vietnam; 2 Hanoi University of Civil Engineering, 55 Giai Phong Street, Hai Ba Trung, Hanoi, Vietnam; 3 Hanoi Medical University, 1 Ton That Tung Street, Dong Da, Hanoi, Vietnam; 4 College of Engineering and Computer Science, VinUniversity, Vinhomes Ocean Park, Gia Lam, Hanoi, Vietnam

**Keywords:** SARS-CoV-2, COVID-19 severity, Vietnam, Clade, PRS

## Abstract

**Background:**

A global pandemic has been declared for coronavirus disease 2019 (COVID-19), which has serious impacts on human health and healthcare systems in the affected areas, including Vietnam. None of the previous studies have a framework to provide summary statistics of the virus variants and assess the severity associated with virus proteins and host cells in COVID-19 patients in Vietnam.

**Method:**

In this paper, we comprehensively investigated SARS-CoV-2 variants and immune responses in COVID-19 patients. We provided summary statistics of target sequences of SARS-CoV-2 in Vietnam and other countries for data scientists to use in downstream analysis for therapeutic targets. For host cells, we proposed a predictive model of the severity of COVID-19 based on public datasets of hospitalization status in Vietnam, incorporating a polygenic risk score. This score uses immunogenic SNP biomarkers as indicators of COVID-19 severity.

**Result:**

We identified that the Delta variant of SARS-CoV-2 is most prevalent in southern areas of Vietnam and it is different from other areas in the world using various data sources. Our predictive models of COVID-19 severity had high accuracy (Random Forest AUC = 0.81, Elastic Net AUC = 0.7, and SVM AUC = 0.69) and showed that the use of polygenic risk scores increased the models’ predictive capabilities.

**Conclusion:**

We provided a comprehensive analysis for COVID-19 severity in Vietnam. This investigation is not only helpful for COVID-19 treatment in therapeutic target studies, but also could influence further research on the disease progression and personalized clinical outcomes.

**Supplementary Information:**

The online version contains supplementary material available at 10.1186/s12879-022-07415-1.

## Introduction

The novel coronavirus disease 2019 (COVID-19) is a respiratory illness caused by severe acute respiratory syndrome coronavirus 2 (SARS-CoV-2). COVID-19 was first reported as an outbreak in Wuhan, China, and proceeded to spread worldwide, resulting in the declaration of a pandemic. The presentation of COVID-19 can range from mild symptoms of fever, cough, headache, muscular pain, nausea, and vomiting to a severe illness characterized by pneumonia, acute respiratory distress syndrome, septic shock, and multi-organ failure [1]. COVID-19 continues to spread around the world, with over 234 million cases and almost 4.8 million deaths as of October 4th, 2021 according to Johns Hopkins university [2].

The ongoing fourth wave of COVID-19 infections in Vietnam is more serious than the previous three. According to the Vietnam Ministry of Health, despite drastic action, Ho Chi Minh City and other southern provinces of Vietnam in particular were still facing complex COVID-19 outbreaks, with more negative impacts on daily life and socio-economic development than in the previous waves. According to the report to WHO, from 3 January 2020 to 5:54pm CEST, 1 October 2021, Vietnam has 790,755 confirmed cases of COVID-19 with 19,301 deaths.

In term of genomic organization, SARS-CoV-2 genome sequence is approximately 27–30 kb in length. This includes two large genes—ORF1a and ORF1b—which encode 16 non-structural proteins (NSP1–NSP16), as well as genes encoding structural proteins S, E, M, and N. One mutation, D614G, is known to have first emerged in the spike protein S, which is responsible for the attachment of the virus to angiotensin-converting enzyme 2, the receptor for SARS-CoV-2 entry into human cells. This European origin variant was dominant in Vietnam in the early March 2020 [3]. There is also evidence of mutations in the receptor-binding domain of the S protein, which are of very high concern given that they can directly influence viral infectivity, transmissibility, and resistance to neutralizing antibodies and T cell responses [4]. Some variants rise rapidly in frequency and then collapse and disappear, while others rise and overtake the dominant strain. Examples of these include B.1.1.7 (United Kingdom variant), B.1.351 (South African variant), B.1.1.28 (Brazilian variant), and B.1.617.2 (Indian variant) [5, 6].

In the blood atlas of COVID-19 hallmarks, Ahern et al. indicated several factors beneficial to the treatment of severe COVID-19 patients, including glucocorticoids (dexamethasone), inhibitors of the IL-6 receptor (tocilizumab/sarilumab), and Janus kinases (baricitinib) [7–11]. Blood-derived signatures that are associated with the disease’s severity are immune suppression, myeloid dysfunction, lymphopenia, interferon-driven immunopathology, T cell activation/exhaustion, and immune senescence [12–17]. In lung tissue, signs include neutrophil and macrophage infiltration, T cell cytokine production and alveolitis, as well as altered redox balance, endothelial damage, and thrombosis [18]. In addition, treatment of patients with corticosteroids, intravenous immunoglobulin, and selective cytokine blockades (tocilizumab) have been associated with higher risk of severe disease [19–21].

A recent study reported 13 genome-wide significant loci that are associated with SARS-CoV-2 infection or severe manifestations of COVID-19. Several of these loci correspond to previously documented associations with lung, autoimmune, and inflammatory diseases [22]. Downes et al. 2021 indicates LZTFL1 as a candidate effector gene at a COVID-19 risk locus in South Asian [23]. Prognostic factors combined with predictive risk models could lead to differentiation of COVID-19 patients based on their risk of severe disease or death. This risk stratification may subsequently guide better disease treatment and personalized outcomes [24]. A polygenic risk score (PRS) that aggregates the information of many common single-nucleotide polymorphisms (SNPs) weighted by the effect size obtained from large-scale discovery genome-wide association study (GWAS) is expected to improve the predictive power and performance of COVID-19 risk assessment [25, 26]. PRS using gene-panel SNPs to calculate associated risk is discussed [27].

**Fig. 1:**
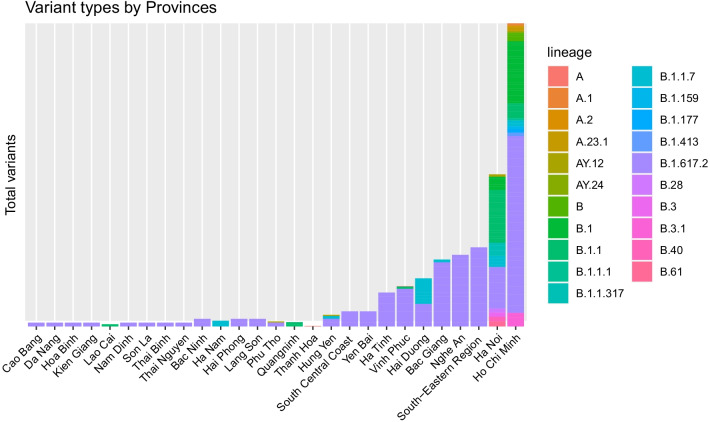
Clade Pango lineage of 361 SARS-CoV-2 samples collected in Vietnam. The Delta variant (B.1.617.2) was the most prevalent variant as of GISAID data collection

**Fig. 2:**
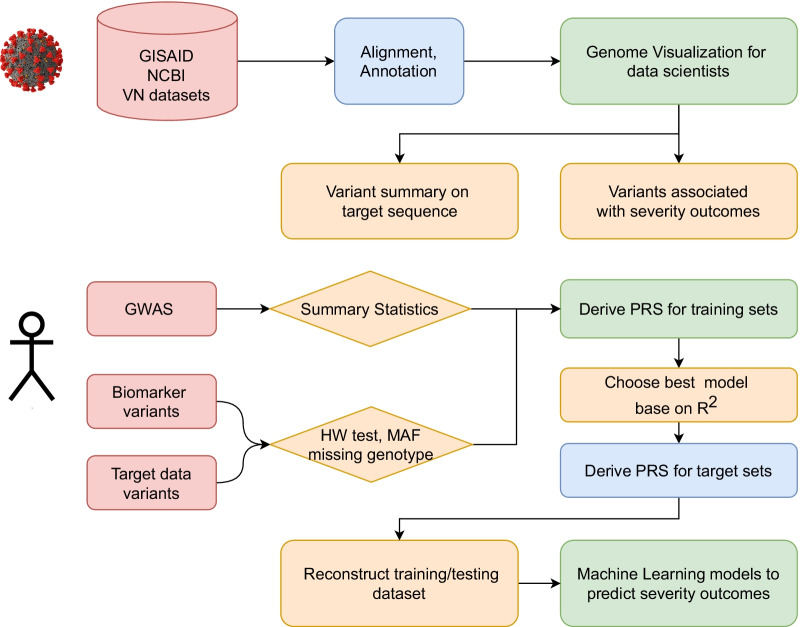
Two workflows have been developed. The first takes as input virus target sequence data from GISAID, the NCBI, and data collected in Vietnam (VN dataset) to identify the virus genome sequence variants and provide summary statistics of these sequences. The second integrates PRSs from two sources including GWAS and a combination of immune biomarker variants associated with the severity of COVID-19 patients

## Materials and methods

### Data processing

Two workflows including a framework to align and annotate SARS-CoV-2 and a predictive model for COVID-19 patients have been developed. The first takes as input virus target sequence data from GISAID, the NCBI, and data collected in Vietnam to identify the virus genome sequence variants and provide summary statistics of these sequences. The second integrates PRSs into machine learning models from two sources: (1) GWAS with hospitalized COVID-19 patients and (2) a combination of immune biomarker variants that are associated with severity status and target data.

The data downloaded from GISAID in Vietnam consisted of 361 SARS-CoV-2 samples, shown in Fig. 1. NCBI data and data collected from Vietnamese sites contained FASTA sequences of SARS-CoV-2, a summary of protein mutations, and patient metadata. Other datasets from different countries in Mekong regions were also downloaded. Nextclade (https://docs.nextstrain.org) is a tool that helps to identifies the differences between target sequences and a reference sequence by Nextstrain to assign clades to these sequences [28]. Nextclade was used for alignment to reference SARS-CoV-2 Wuhan-1 (MN908947.3), and for detecting variants and protein mutations on these datasets.

With data from NCBI, we have gathered 322,101 samples collected in Q2 2021 (Quarter 2 of 2021) and 542,275 samples collected in Q3 2021. All samples had a length greater than 29,000 bases and number of Ns is less than 300. These samples were also analyzed by Nextclade to find out which strains and mutations prevail among others.

COVID-19 data from 57,560 patients across 63 Vietnamese provinces (approximately as of July 20, 2021), and other data from almost 19,924 patients were downloaded from public source [29]. Age, sex, status, and other metadata of each patient were included. The patient’s province of residence was a crucial parameter in the model as it represents the environment of coronavirus disease.

Dataset from Whole-Genome Sequencing from Vietnam in The International Genome Sample Resource (IGSR) [30] will be used in the study. The target dataset is a cohort of 99 (of 124 samples) unrelated Vietnamese people in the project (whole-genome sequencing with 30× coverage) from Kinh ethnic group (100 KHV) on GRCh38 [31]. The $\chi ^2$ goodness-of-fit test for Hardy–Weinberg equilibrium was used on samples with related individuals, and missing genotypes were filtered. We also use the dataset from 1000 Vietnamese Genomes Project (1KVG), a source of genomic variants for Vietnamese population by sequencing the whole genome of 1008 unrelated healthy Vietnamese to a depth of at least 28× [32].

The most common method for calculating PRS is called clumping and thresholding (or pruning and thresholding), applies two filtering steps as shown in Fig. 2. SNPs that weakly correlated with each other were retained. Clumps around SNPs were formed by using the linkage disequilibrium clumping procedure [33].

In PRS analyses can be characterized by the two input data sets: (i) base (GWAS) data: summary statistics (e.g. betas, p-values) of genotype-phenotype associations at genetic variants and (ii) target data: genotypes and phenotype(s) in individuals of the target sample [34]. We investigated the blood and lung biomarkers incorporated into the model for 100 KHV and derived PRS for all individuals. Based on the result, we will reconstruct PRS for a larger dataset and apply several machine learning techniques to predict severity of COVID-19 patients in Vietnam.

### Integrating biomarkers and target data variants into PRS computation

GWAS summary statistics of COVID-19 patient variants of Hospitalized vs. not hospitalized were downloaded. The summary was thresholded at $p = 5e-8$ as standard in QC of GWAS. Summary statistics of COVID-19 were downloaded from open source COVID-19 HGI GWAS (https://www.covid19hg.org). These summary statistics are the result of a meta-analysis of 61 studies from 24 countries, and include the weights (effect sizes) and p-values of 13,498,845 variants, derived from a genotype-phenotype association study with 14,480 hospitalized patient samples and 73,191 control samples. GWAS QC excluded variants with p-value greater than 0.05. The PRS was derived for the training set using a pruning and thresholding method by Plink v1.9 [35]. The best model was selected based on $R^2$. The PRS was calculated by the below equation of Plink.

1 $$\begin{aligned} PRS_j = \frac{\sum _i^N {S_i} * G_{i_j}}{P * M_j} \end{aligned}$$

The effect size of SNP *i* is $S_i$, effect allele *j* is $G_{ij}$, the ploidy of the sample is *P* (with humans, $P = 2$), the number of SNPs is *N*, the number of non-missing SNPs in sample *j* is $M_j$. In addition, individual phased genotype data of 100 KHV was from a VCF file. Standard quality controls were applied to the KHV VCF files, with missing genotype $> 0.1$, Hardy–Weinberg Equilibrium $P > 1e^{-6}$, minor allele frequency $MAF < 0.01$.

### Reconstructing training and testing data for machine learning models

The lack of direct PRS calculations for patients from Vietnam without a genotyping/sequencing profile posed a major challenge. Instead of directly predicting PRS using existing methods, we used a reconstruction method that applies a multivariate linear model to use the PRS calculations of an existing cohort (reference matrix *Ref* with PRS) with genotyping/sequencing to other cohorts, and showed the model can improve the prediction of severity. The model utilizes covariates captured age, gender, location. The predicted PRSs for 19,924 COVID-19 patients were then derived by a machine learning model to predict severity, and that correlated well with the measurements from clinical readouts.

2 $$\begin{aligned} ReconstructPRS = \sum _{i}^{N} Fraction^i_j \times PRS^j_{Ref} \end{aligned}$$

*ReconstructPRS* is reconstructed PRS using the summation of all fraction ($Fraction^i_j$) measured by a covariate *i* and a sample *j* in the reference cohort and PRS of sample *j* in the cohort $PRS^j_{Ref}$. *N* is the number of covariates.

SNPs relevant to COVID-19 were then ranked by probability of severity according to a COVID-19-related study from the GWAS catalog (downloaded in August 2021 from https://www.ebi.ac.uk/gwas/). The data show that provinces are highly correlated across datasets. Since the sequencing data of these 19,924 patients is not public, we used 100 KHV results to calculate and reconstruct PRS for the training and testing datasets. The PRS was based on GWAS on GRCh38 from [36], using the pruning and thresholding method as mentioned previously. The predictive model showed how certain non-genetic factors may impact the risk of hospitalization due to the virus. We used three machine learning models including SVM, Random Forest and Elastic Net to predict COVID-19 severity in Vietnamese patients based on PRS and other covariates such as age, sex, location, exercise, and underlying conditions. The training dataset contained 11,814 patients, with status of deceased, active and recovered. These statuses were assigned numeric values in the model. To simplify the models, this was converted to a binary class of ‘Active’ and ‘Recovered’. Deceased patients were excluded.

## Result

### Comparative analysis for SARS-CoV-2 sequences by country using NCBI data

**Fig. 3:**
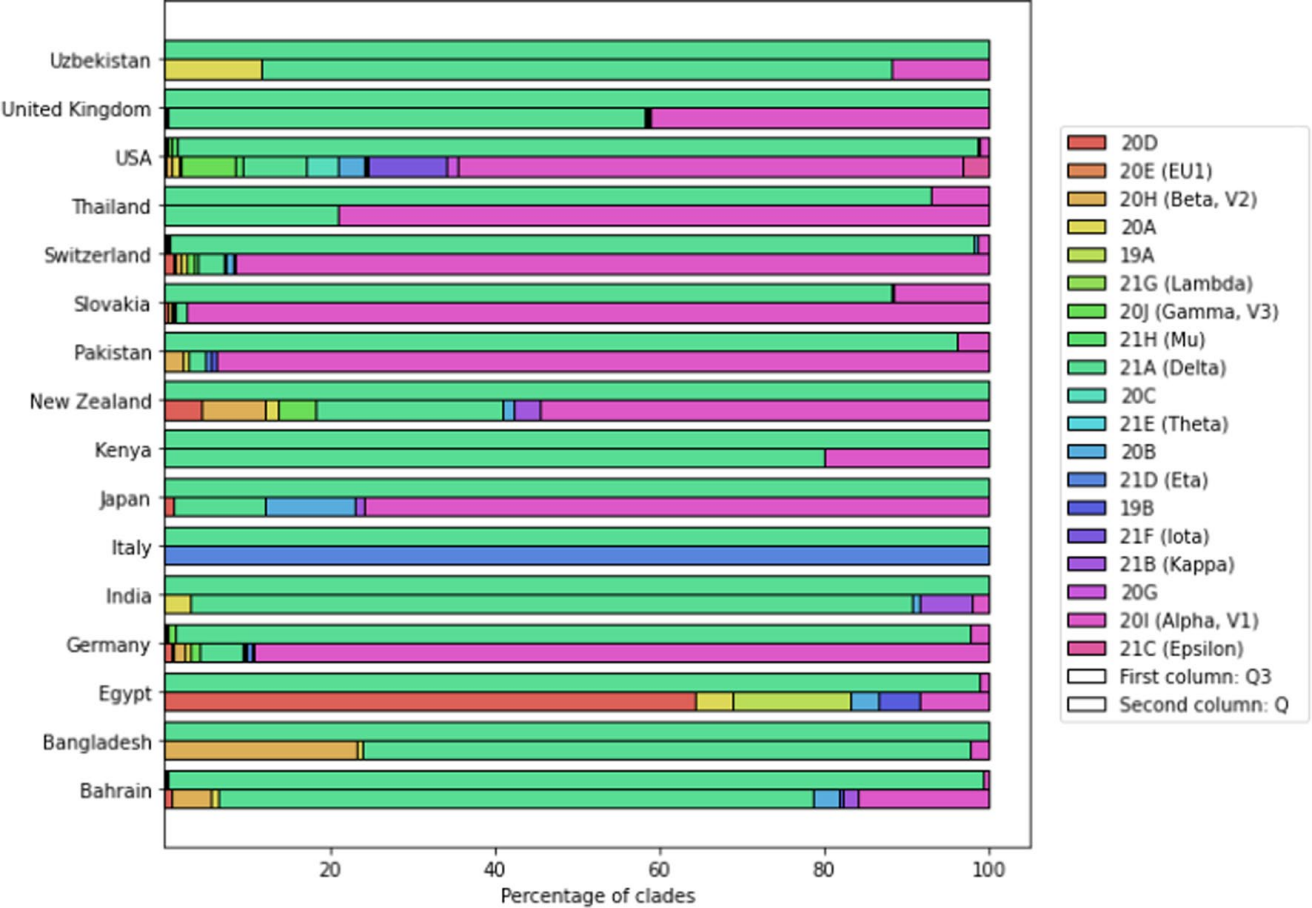
Sequence analysis of SARS-CoV-2 among countries

Figure 3 shows the percentage of SARS-CoV-2 clades in different countries for Q2 and Q3 2021. Overall, the strains in Q2 were quite diverse with common strains such as 20A, 20I (Alpha), 21A (Delta), 20B, 21F (Iota) but in the third quarter, strain 21A (Delta) predominated in most of the countries. In the case of the Delta variant, in the second quarter, a number of Asian countries such as India, Bahrain, Bangladesh, and Uzbekistan recorded the presence of this variant with a significant majority, while some European and American countries such as the US, Switzerland, Germany, this variant appeared but did not prevail. This indicates that the outbreak of the Delta variant took place first in Asian countries, then in European and American countries. Regarding mutations, our analysis results also show that the most common mutations in the third quarter are the typical mutations of the Delta variant such as S:D614G, S:P681R, S:L452R, S:T478,S:R158G. In summary, the data analyzed on NCBI show the emergence and the spread of the Delta variant and its mutations in recent times.

### Comparative analysis for SARS-CoV-2 sequences in Vietnam and Thailand using GISAID data

**Fig. 4:**
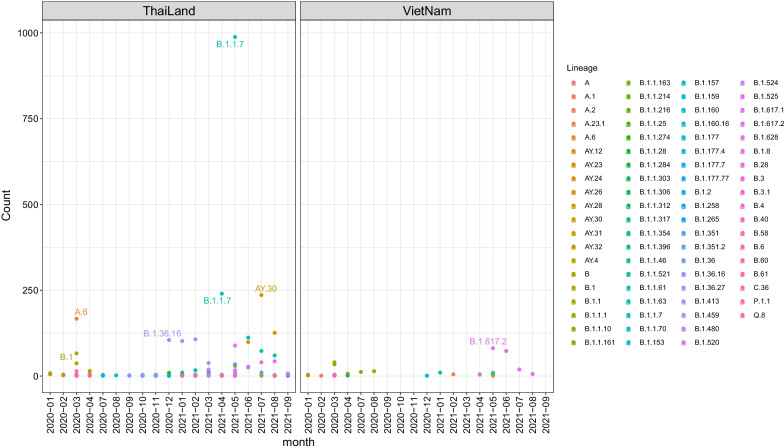
Number of SARS-CoV-2 sequences with different clades from Vietnam and Thailand

In this part, 3211 FASTA sequences from Thailand in GISAID have been used. We compared Vietnam and Thailand populations as they have similar genetic characteristics in other infectious diseases [37] and their data is widely available in Southeast Asia. Comparison of the number of sequences by each month shows that Thailand had a prevalence of Lineage B.1.1.7 (Alpha) in the second quarter of 2021, while Vietnam had a prevalence of Lineage B.1.167.2 (Delta) from May of 2021. In addition, lineage A.6, B.1.36.16 and AY.30, which first appeared in South East Asia, were detected mostly in Thailand (Fig. 4). The analysis is consistent with the result from Chookajorn et al. 2021 [38] as the spread of the Alpha and Delta variants dominant over the region raised serious problems of the healthcare system. As an emerging epicenter of COVID-19 pandemic, Southeast Asian countries needs to take immediate collaborative actions to resolve these problems. More details of the analysis can be found in Additional file 1: Figs. S1 and S2.

### Comparative analysis for SARS-CoV-2 sequences in Vietnam between hospitalized and recovered patients

**Fig. 5:**
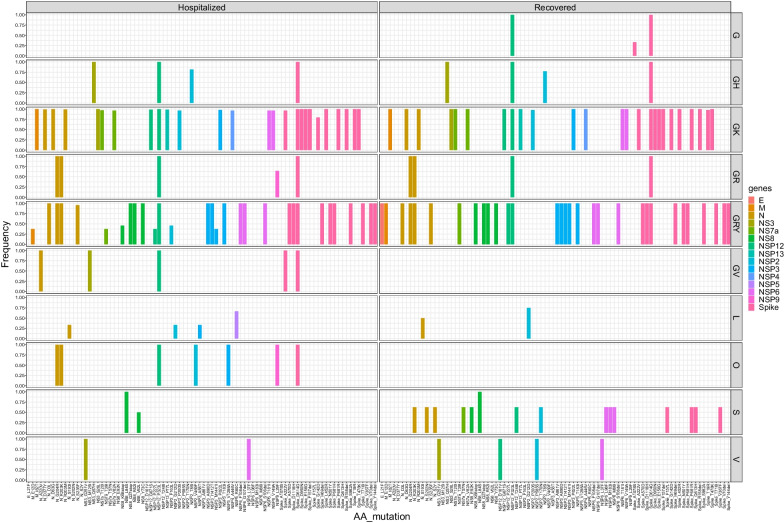
Histogram of individuals by variants in Vietnam of Hospitalized and Recovered COVID-19 patients. Of the currently known SARS-CoV-2 clades, clade GR was the most prevalent worldwide, followed by GV and then GH

In GISAID datasets, there are 361 FASTA sequences of SARS-CoV 2 from Vietnam. The virus variants are divided into 10 Clades (G,GH,GK,GR,GRY,GV,L,O,S,V). More details on GISAID’s clades can be found on the website (https://www.gisaid.org/). The result of comparison between all clades from Vietnam shows two common variants D614G (in Spike region), P323L (in NSP12, known as ORF1a region) in almost all clades with prefix G in both groups of Hospitalized and Recovered patients. These 2 mutations overtake frequency of dominant strain. Furthermore, clade GK and GRY have more protein mutations than other clades that can be promising targets for for analyzing protein structure and designing COVID-19 vaccines or drugs (Fig. 5).

### Biomarker variants and target data variants associated with severity on Vietnamese cohorts

We formed nine gene sets associated with severity of COVID-19 patients, as introduced in "Introduction" section. Table 1 reports the immune gene sets, along with the number of genes in each set and the number of SNPs found in 100 KHV.

**Table 1:** Immune gene sets associated with severity of COVID-19

Geneset/first author	Number of genes	Number of SNPs	References
IL6/Gordon	87	17,653	[7]
Dexamethasone/Horby	19	5322	[9]
Immunesuppression/Bost	3	13,612	[12]
Myeloid dysfunction/Chen	4	534	[13]
Lymphopenia/Diao	69	237	[14]
Interferon immunopathology/Hadjadj	20	3724	[15]
Tcell/Mann	200	34,942	[16]
Immune senescence/SchulteSchrepping	49	12,406	[17]
Endothelial/Grant	13	1584	[18]

Allele frequencies for SNPs of genes in each gene set were calculated for both 100 KHV and 1KVG [32]. SNP allele frequencies for all gene panels were highly correlated between sets (Pearson correlation $p-value < 2.2e^{-16}, R = 0.99$) (Additional file 1: Fig. S3). 1KVG was able to detect some variants with much lower allele frequency compared with those frequencies of 100 KHV suggesting that using 1KVG (with much larger sample size) to increase the quality of variants, especially in immunogenic and drug targets used in Vietnamese people. These variants were added to the model as “causal” SNPs in the computation of PRS as illustrated in the second workflow in Fig. 1.

### Machine learning models to predict severity outcomes in Vietnam

The two datasets from [29] (57,560 patients split by province and 19,924 patients with province information provided) were consistent in the distribution of patients between provinces (Pearson correlation $p = 1.2e^{-15}, R = 0.83$). This is an important result as location and other phenotypes were used to reconstruct PRS in the training and testing datasets for the machine learning models. The data has been divided by training 70% and testing 30% number of samples in the datasets.

**Fig. 6:**
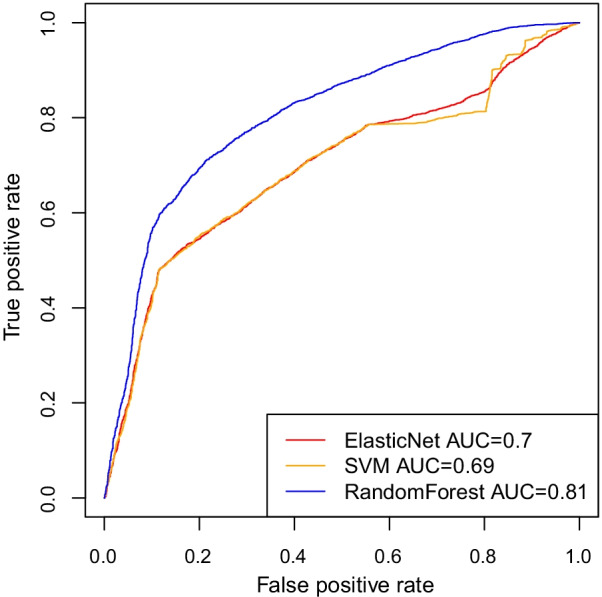
High training accuracy varied by machine learning method to predict severity status of COVID-19 patients based on PRS and other covariates

With an average of 100 runs, Random Forest was the best model with AUC = 0.81, followed by Elastic Net with AUC = 0.7 and SVM with AUC = 0.69 in Fig. 6.

## Discussion

Statistical analysis of the SARS-CoV-2 sequences obtained from the NCBI indicates that the predominant virus variants at a period of time may vary between countries and regions of the world. If the severity of COVID-19 is related to the variation of the virus and the genetics of a population, then this association should be analyzed by country and ethnic group. On the other hand, the statistics for two time periods also show that the dominant strain in a country can change over time, the new dominant variant can replace existing variants. The dynamic change of SARS-CoV-2 variants requires prediction of COVID-19 severity in patients to be performed regularly to stay up to date with current prevailing variants.

In this study, we used the genotype data of 99 samples from the 1000 Genomes Project, which were recruited only from Ho Chi Minh City. Although the Kinh ethnic group is the main ethnic group in Vietnam, accounting for 86% of the country’s population [39], these individuals may not represent the entire Vietnamese population. Therefore, we suggest that further investigation should be carried out with a 1000 Vietnamese Genomes Project dataset [32] recruited from 1008 unrelated individuals across the country, according to population distribution. We would expect this to increase the number of SNPs with allele frequency $> 1\%$. In this dataset, the metadata for these 1008 samples should include not only the basic health indices of BMI, blood pressure, glucose level, cholesterol level, and white blood cell count but also information about any chronic or hereditary diseases, as well as allergy factors (foods, drugs, or insects) and lifestyle factors (alcohol, cigarettes). These factors also influence the health and resilience of an individual against SARS-CoV-2 infection.

In addition to immune profiling, the prediction of COVID-19 severity in patients requires the evaluation of factors such as underlying disease [40], vaccination status, and the patient’s intrinsic genetic response or adverse reactions to some drugs, especially some antibiotic therapies used for bacterial co-infection at ICU admission [41]. Allergy to $\beta$-lactam drugs like penicillin or amoxicillin, mainly caused by genetic factors from the interleukin and Human Leukocyte Antigen systems, is highly prevalent according to the National Centre of Drug Information and Adverse Drug Reactions [42]. We have studied numerous COVID-19 drugs, especially some used in Vietnam for COVID-19 outpatients and their PharmGKB IDs [43, 44] (Dexamethasone—PA449247, Methylprednisolone—PA450466, Prednisolone—PA451096, Rivaroxaban—PA165958360, Apixaban—PA166163740, and Remdesivir—PA166197141) (4109/QƉ-BYT issued by Vietnam Ministry of Health on August 26, 2021) that have allele frequency (for target gene variants in each drug) in 100 KHV in Additional file 1: Fig. S4. This further investigation can be useful for the treatment benefit of Vietnamese patients when in hospital.

We initiated an effort to study the relationship between immunogenic profiling and SARS-CoV-2 infection severity by incorporating PRS based on immune gene sets. This approach is comprehensive as it incorporates PRS and immunogenic profiling of Vietnamese people. While providing novel scientific insights in Vietnam remains a major priority of this initiative study, we equally value learning from and collaborating with other countries in the Mekong regions (Cambodia, Laos, Myanmar, and Thailand) and other countries around the world. We expect to substantially contribute to the understanding of the variability of COVID-19 severity in Vietnam (Additional files 2, 3, 4, 5, 6, 7).

## Conclusion

In this paper, we have investigated the SARS-CoV-2 profiling in Vietnam using various data sources and a predictive model of COVID-19 severity, using immunogenic profiling of the Vietnamese population based on investigation of SNPs in GWAS and metadata from 124 Vietnamese people (KHV) in the 1000 Genomes Project. Machine learning models showed high accuracy in predicting the hospitalization status of a very large dataset of Vietnamese COVID-19 patients. We expect to improve our model by using 1KVG dataset with both novel and known variants in order to have a better understanding of the immunogenic profiling of Vietnamese people. This initial approach will not only be helpful in understanding susceptibility to SARS-CoV-2 infection, but could also inform how to control the disease, as well as treatment progression and recovery. By this way, we hope to make an impact on human health and healthcare systems in the areas of Vietnam affected by COVID-19 pandemic.

## Supplementary Information

**Additional file 1: Fig. S1.** Percentage of Clades by month in Vietnam and Thailand. **Fig. S2.** Percentage by Lineage by month in Vietnam and Thailand. **Fig. S3.** Scatter plots of allele frequency from datasets: one from WGS of Vietnamese people in the 1000 Genomes Project with high coverage, and the other from WGS of 1000 Vietnamese people [1] with nine immune gene sets associated with severity of COVID-19 in Vietnam. **Fig. S4.** Scatter plots of allele frequency from datasets: one from WGS of Vietnamese people in the 1000 Genomes Project with high coverage, and the other from WGS of 1000 Vietnamese people [1] with 6 target gene sets of COVID-19 drugs used in Vietnam.

**Additional file 2. Table S1.** SarsCov2Q2Q3: The number of sequences included in the Q3 and Q2 columns for each country.

**Additional file 3. Table S2.** IL6 Gordon: The overlapping SNPs between IL6 gene set (Gordon et al.) with GWAS significant set (e.g., p-value < 5e-8).

**Additional file 4. Table S3.** Myeloid dysfunc Chen: The overlapping SNPs between myeloid dysfunction gene set (Chen et al.) with GWAS significant set.

**Additional file 5. Table S4.** Interferon Hadjadj: The overlapping SNPs between Interferon gene set (Hadjadj et al.) with GWAS significant set.

**Additional file 6. Table S5.** Tcell Mann: The overlapping SNPs between Tcell gene set (Mann et al.) with GWAS significant set.

**Additional file 7. Table S6.** All genset: The overlapping SNPs between all gene set with GWAS significant set.

## Data Availability

Our wrapped pipeline for generating Polygenic Risk Score and all processed data are available for public use at https://github.com/VMGiang/SeverityCOVID19.
