# Supplementary material for: Investigation of target sequencing of SARS-CoV-2 and immunogenic GWAS profiling in host cells of COVID-19 in Vietnam
Source: BMC Infect Dis. 2022 Jun 19;22:558. doi: 10.1186/s12879-022-07415-1 (PMC9206857; doi:10.1186/s12879-022-07415-1)
Supplement: Supplementary file 1 — Additional file 1: Fig. S1. Percentage of Clades by month in Vietnam and Thailand. Fig. S2. Percentage by Lineage by month in Vietnam and Thailand. Fig. S3. Scatter plots of allele frequency from datasets: one from WGS of Vietnamese people in the 1000 Genomes Project with high coverage, and the other from WGS of 1000 Vietnamese people [1] with nine immune gene sets associated with severity of COVID-19 in Vietnam. Fig. S4. Scatter plots of allele frequency from datasets: one from WGS of Vietnamese people in the 1000 Genomes Project with high coverage, and the other from WGS of 1000 Vietnamese people [1] with 6 target gene sets of COVID-19 drugs used in Vietnam. [file 12879_2022_7415_MOESM1_ESM.pdf]

BMC infectious diseases supplementary

Tham Hoang

November 2021

List of Supplementary Figures

1 Percentage of Clades by month in Vietnam and Thailand. 2
2 Percentage by Lineage by month in Vietnam and Thailand. . . . 2
3 Scatter plots of allele frequency from datasets: one from WGS of Vietnamese people in the 1000 Genomes Project with high coverage, and the other from WGS of 1000 Vietnamese people [1] with nine immune gene sets associated with severity of COVID-19 in Vietnam. 3
4 Scatter plots of allele frequency from datasets: one from WGS of Vietnamese people in the 1000 Genomes Project with high coverage, and the other from WGS of 1000 Vietnamese people [1] with 6 target gene sets of COVID-19 drugs used in Vietnam. . . 4

1 Supplementary Figures

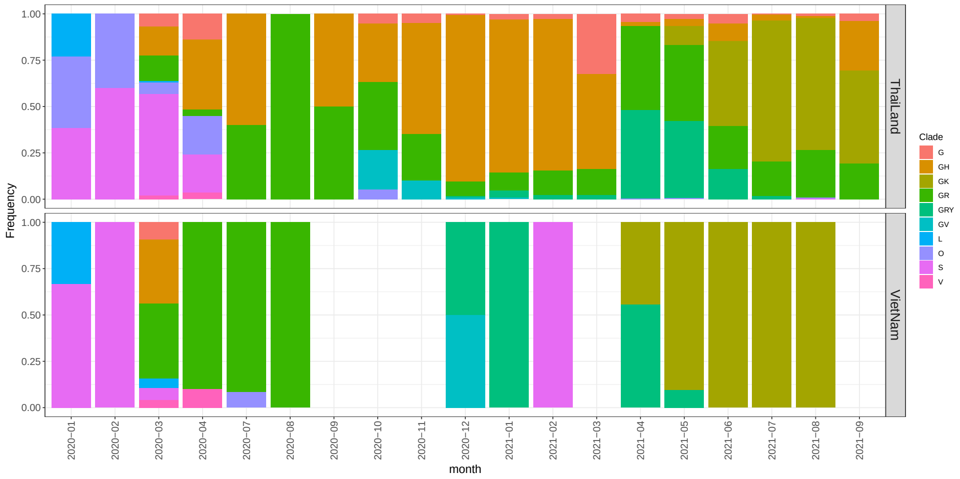

Supplementary Figure 1: Percentage of Clades by month in Vietnam and Thailand.

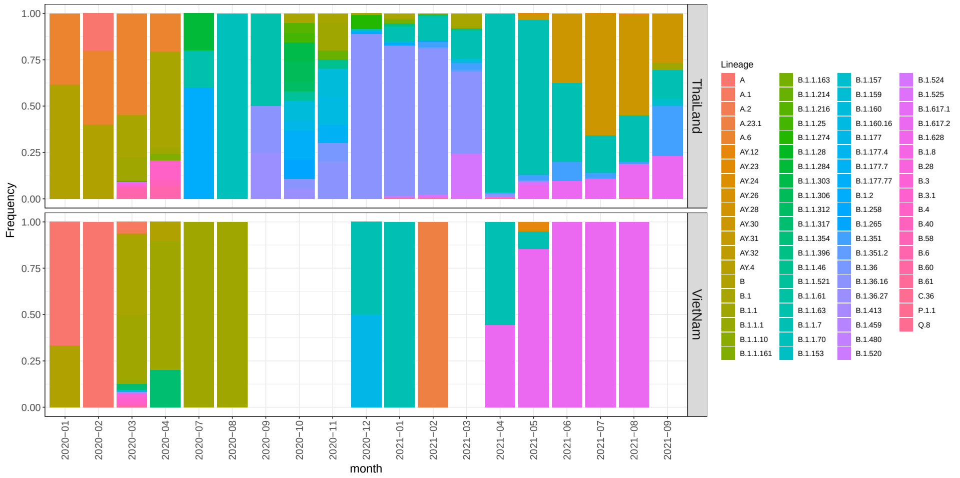

Supplementary Figure 2: Percentage by Lineage by month in Vietnam and Thailand.

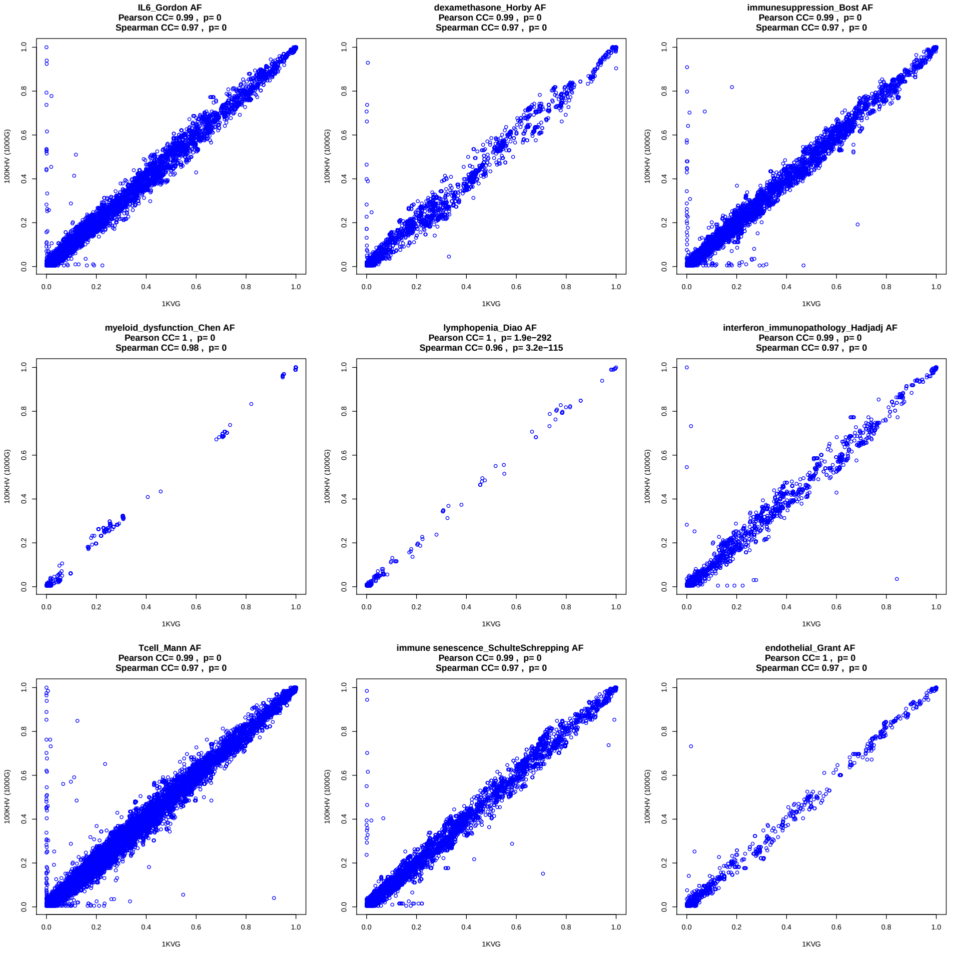

Supplementary Figure 3: Scatter plots of allele frequency from datasets: one from WGS of Vietnamese people in the 1000 Genomes Project with high coverage, and the other from WGS of 1000 Vietnamese people [1] with nine immune gene sets associated with severity of COVID-19 in Vietnam.

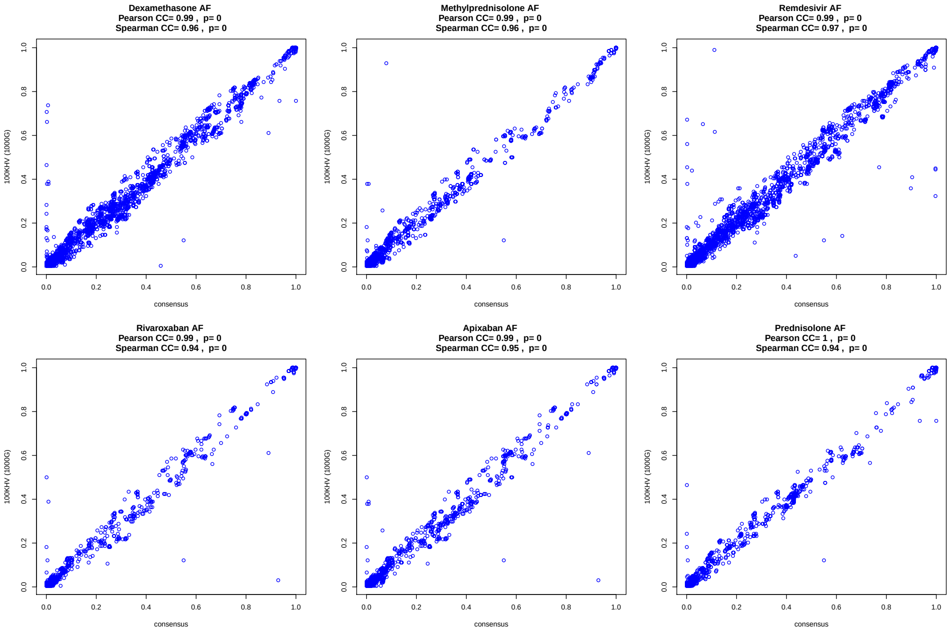

Supplementary Figure 4: Scatter plots of allele frequency from datasets: one from WGS of Vietnamese people in the 1000 Genomes Project with high coverage, and the other from WGS of 1000 Vietnamese people [1] with 6 target gene sets of COVID-19 drugs used in Vietnam.

References

[1] 1kvg data portal. https://genome.vinbigdata.org/.
